# Recurrent inferior shoulder subluxation secondary to incidental traumatic plexitis following arthroscopic rotator cuff repair

**DOI:** 10.1016/j.xrrt.2026.100754

**Published:** 2026-04-09

**Authors:** Chaiyanun Vijittrakarnrung, Theeranop Temtheerakij, Peerapat Lertwiram

**Affiliations:** Department of Orthopedics, Faculty of Medicine Ramathibodi Hospital, Mahidol University, Bangkok, Thailand

**Keywords:** Arthroscopic rotator cuff repair, Inferior shoulder subluxation, Brachial plexitis, Delayed complication, Double-crush syndrome, Electromyography

## Introduction

Peripheral neuropathies are an uncommon complication of shoulder arthroscopy, with an overall complication rate of approximately 1%.^10^ The multiple etiologies of peripheral nerve injury include prolonged arm positioning, traction, compression, gravity, edema from arthroscopic fluid, and sling immobilization. While spontaneous resolution occurs in the majority of patients, some experience persistent neuropathic symptoms.^19^ Axillary nerve palsy is one of the most common nerve injuries associated with shoulder arthroscopy,^7^ frequently manifesting as deltoid atony, which can lead to inferior subluxation of the humeral head.

The pathophysiology of inferior humeral head subluxation involves a complex interplay of muscle fatigue, capsular injury, loss of negative intra-articular pressure, and neural compromise. These mechanisms often manifest following acute events like glenohumeral dislocations and proximal humeral fractures.^12^^,^^20^^,^^24^ In the post-operative setting, subluxation is notably prevalent, affecting up to 42% of patients within 2 weeks of fracture fixation.^15^ However, distinguishing structural etiologies from neuropathic causes is difficult, particularly when the presentation does not align with typical post-operative timelines.

While current literature predominantly describes nerve injuries as immediate complications,^5^^,^^6^^,^^11^^,^^16^ we report a case characterized by a delayed onset of inferior subluxation occurring 3 weeks after arthroscopic rotator cuff repair.

## Case report

**Figure 1:**
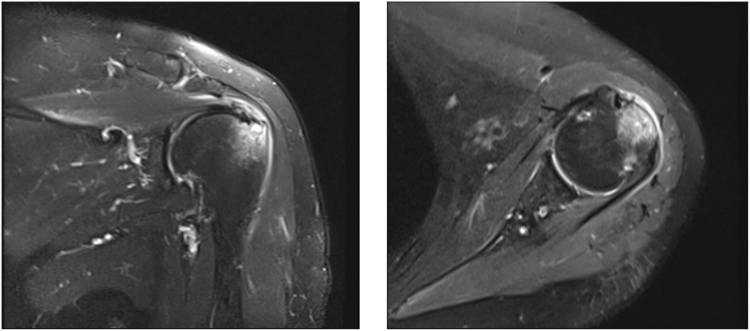
Pre-operative MRI of the left shoulder showing a high-grade partial-thickness bursal-sided supraspinatus tear and subacromial-subdeltoid bursitis. *MRI*, magnetic resonance imaging.

A 61-year-old Thai female presented with a 6-month history of chronic left shoulder pain and weakness. The pain was exacerbated by lifting objects or reaching end-range motion. Her medical history included hypertension and dyslipidemia. She denied antecedent trauma, neck pain, or prior neurological deficits. Physical examination revealed a painful arc, limited range of motion, and positive results on Jobe's and resistant external rotation stress tests; the pre-operative neurological examination was intact. Magnetic resonance imaging (MRI) of the left shoulder demonstrated supraspinatus tendinosis with a 1.1 × 0.7 cm high-grade bursal-sided tear and subacromial-subdeltoid bursitis (Fig. 1). The patient was diagnosed with subacromial impingement and a supraspinatus tear.

**Figure 2:**
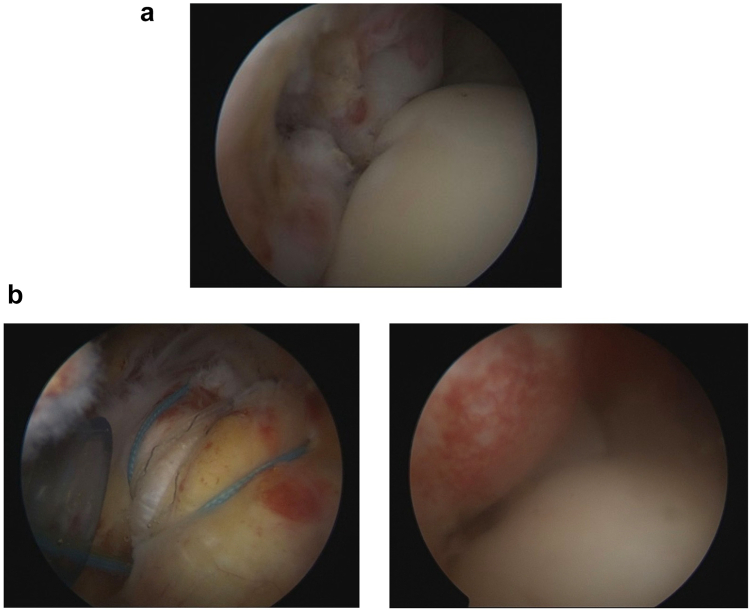
(**a**) Intraoperative intra-articular arthroscopic view of the left shoulder (viewed from the posterior portal) demonstrating a full-thickness tear of the supraspinatus tendon. (**b**) Arthroscopic images showing the final rotator cuff repair from both intra-articular and extra-articular (bursal side) perspectives.

Following failed conservative treatment, the patient underwent arthroscopic rotator cuff repair (ARCR) in the beach chair position under general anesthesia and a superior trunk interscalene block. The procedure was performed with an arthroscopic fluid pressure maintained at 40 mmHg, utilizing a total of 3,000 mL of irrigation fluid. The operative time was 65 minutes. Although the pre-operative MRI indicated a high-grade partial-thickness tear of the supraspinatus tendon, intraoperative visualization revealed a complete full-thickness tear (Fig. 2*A*). There was also a subacromial spur and a degenerative superior labrum anterior and posterior type 2 lesion with biceps fraying. The supraspinatus was repaired using a double-row transosseous-equivalent technique (Fig. 2*B*), accompanied by biceps tenotomy and acromioplasty. Post-operatively, the shoulder was immobilized in a standard arm sling without an abduction pillow. Regarding the post-operative course, the patient was admitted for overnight observation and discharged on post-operative day 1. A neurological assessment conducted 12 hours post-operatively confirmed that the superior trunk interscalene block remained partially intact, with expected gradual dissipation; the total duration of the block was approximately 24 hours. Thereafter, the patient remained neurologically intact, with preserved deltoid tone and sensation, and was discharged without immediate complications.

**Figure 3:**
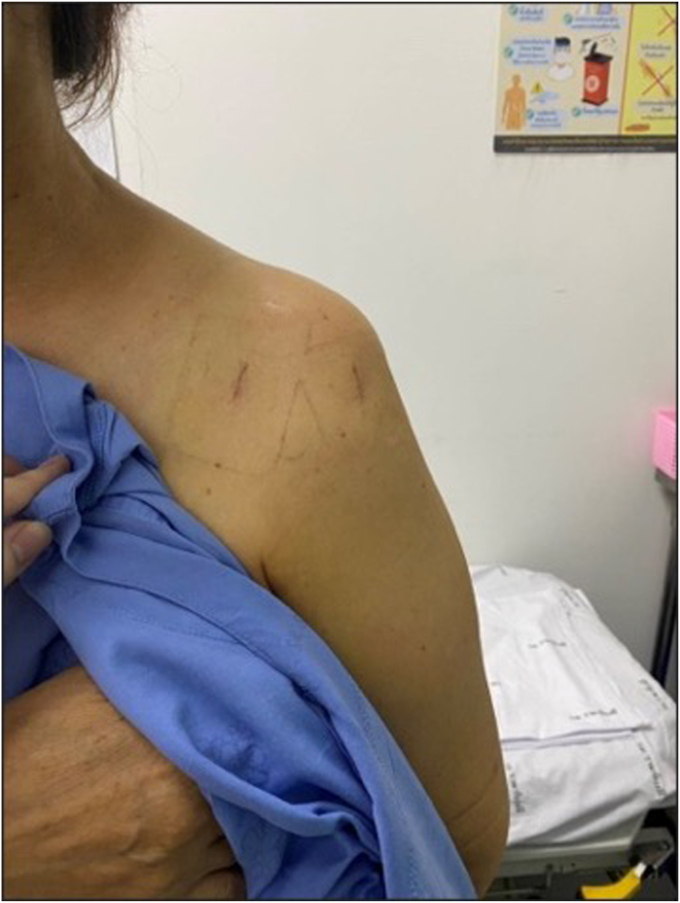
Clinical photograph of the patient's left shoulder at three weeks postsurgery, demonstrating a loss of deltoid contour (positive Ruler sign).

**Figure 4:**
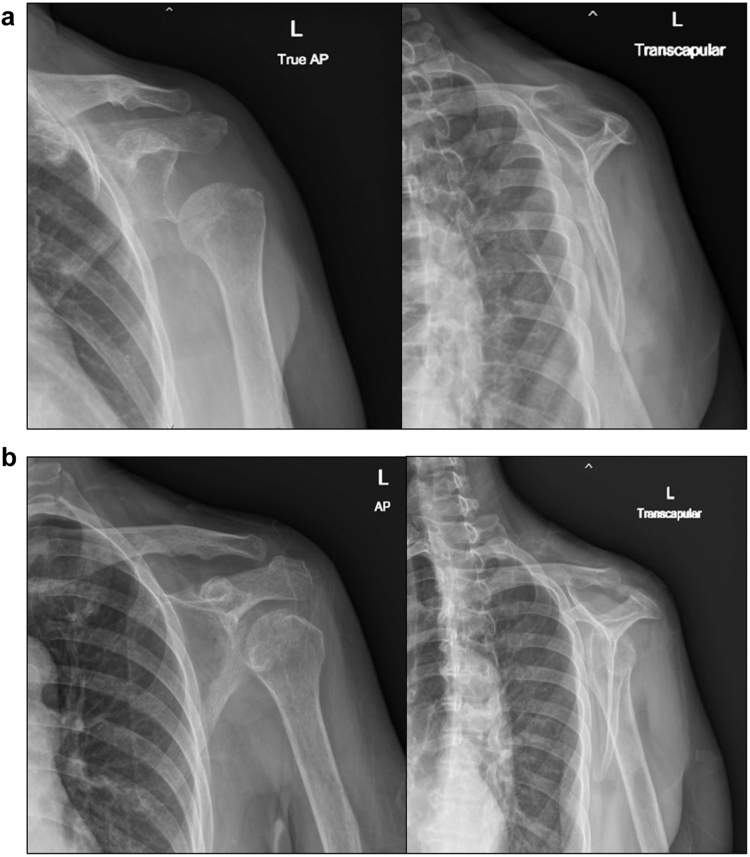
(**a**) Pre-reduction plain radiograph showing inferior subluxation of the *left* humeral head. (**b**) Postreduction plain radiograph confirming restored glenohumeral alignment. *AP*, anteroposterior.

Rehabilitation commenced on the first post-operative day with pendulum exercises and passive flexion to 90°, as well as a protocol for 6 weeks of sling immobilization. At the 2-week follow-up, the incision was well-healed, and no neurological deficit was noted. However, at 3 weeks postsurgery, the patient experienced sudden onset pain radiating from the left shoulder to the elbow, accompanied by a mechanical “clunk” while stretching her neck during bathing. She presented to the emergency room, where physical examination revealed a positive Ruler sign (absence of deltoid contour) (Fig. 3) and decreased sensation in the C5-C6 dermatomes. Notably, motor function of the median, radial, and ulnar nerves remained intact. Plain radiographs confirmed inferior subluxation with an anterior translation component (Fig. 4*A*). Due to concerns regarding the integrity of the recent rotator cuff repair, a manual reduction using the “zero maneuver” was performed in the emergency department under conscious sedation (5 mg of diazepam and 3 mg of morphine) (Fig. 4*B*) and immobilized in an arm sling. This gentle technique was chosen to avoid excessive torque on the repaired supraspinatus. A palpable sense of relocation was achieved during the procedure. This intervention was aimed at restoring joint congruity and, more importantly, alleviating acute mechanical tension on the brachial plexus caused by the subluxation.

**Figure 5:**
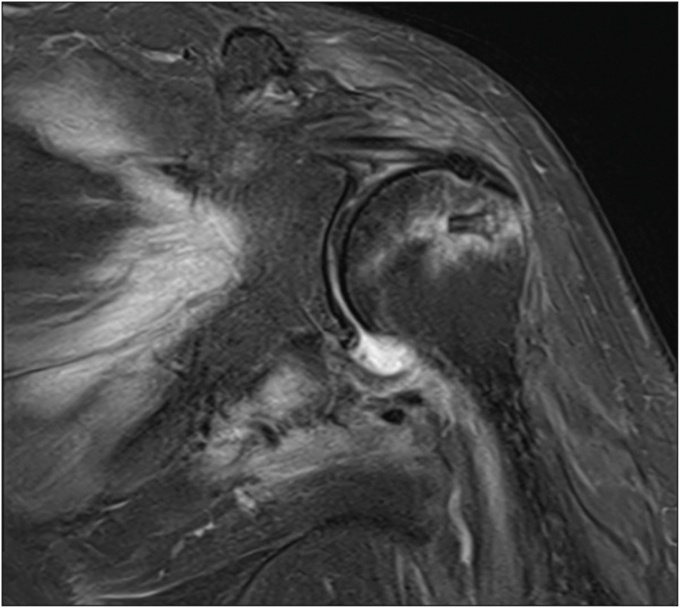
Post-reduction MRI of the left shoulder showing no evidence of rotator cuff re-tear, confirming the integrity of the initial repair. *MRI*, magnetic resonance imaging.

**Figure 6:**
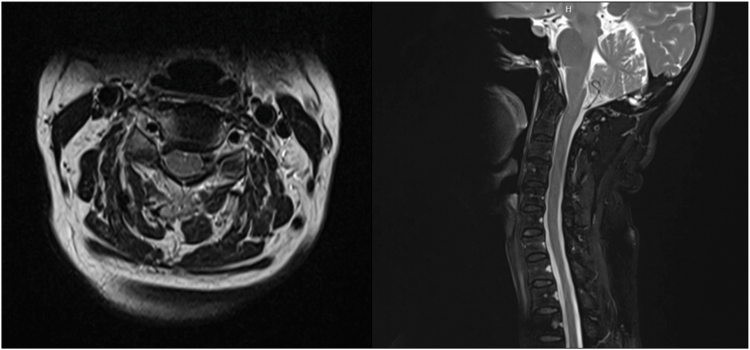
Sagittal and axial MRI views of the cervical spine demonstrating mild spondylosis and canal stenosis without evidence of acute C5-C6 spinal cord or nerve root compression. *MRI*, magnetic resonance imaging.

The patient returned the following day with recurrent inferior subluxation. She was readmitted for reduction and stabilized in a Bobath sling. This specialized shoulder orthosis is designed to counteract inferior subluxation by providing upward support to the humerus, thereby realigning the humeral head within the glenoid fossa. Unlike standard slings, it stabilizes the shoulder proximally while allowing for distal limb mobility and functional use during the recovery of the paralyzed muscles. Repeated shoulder MRI showed no evidence of rotator cuff retear (Fig. 5). Cervical spine MRI revealed mild spondylosis and canal stenosis but, crucially, no severe compression of the C5-C6 spinal cord or nerve roots (Fig. 6).

The definitive diagnosis was established via comprehensive electrodiagnostic evaluation. Electromyography (EMG) revealed acute denervation of the left biceps brachii, deltoid, and infraspinatus muscles. To further localize the lesion, peripheral magnetic stimulation (PMS) was performed using a high-intensity magnetic stimulator with a circular coil placed over the C5-C6 paraspinal area and the Erb's point. Unlike conventional electrical stimulation, PMS can achieve deep tissue penetration without the discomfort associated with high-voltage electricity, allowing for the assessment of proximal nerve segments. In this patient, PMS demonstrated a significant reduction in compound muscle action potential amplitudes and prolonged latencies in the deltoid and biceps, specifically involving the left C5 nerve root, consistent with the acute denervation observed on needle EMG. These findings localized the pathology to the proximal brachial plexus and cervical root levels, distinguishing the condition from a simple peripheral nerve injury. The final diagnosis was incomplete brachial plexitis involving C5-C6.

**Figure 7:**
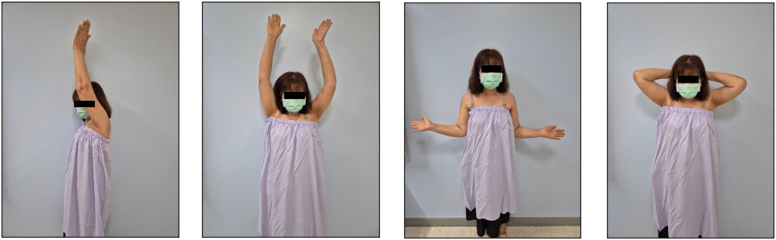
Clinical photograph at three months postinjury showing full restoration of active range of motion.

**Figure 8:**
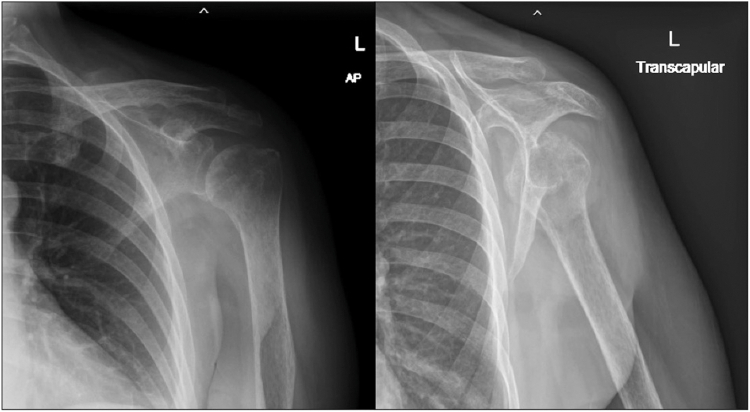
Follow-up plain radiographs showing maintained reduction and normal alignment of the glenohumeral joint, with no residual inferior subluxation. *AP*, anteroposterior.

Due to immediate recurrence following multiple closed reduction attempts, manual reduction was abandoned. The patient was managed conservatively with an arm sling, physical therapy, and medication to address moderate radiating pain and chronic dull shoulder ache. Four weeks later, despite persistent symptoms, repeat EMG indicated signs of muscle reinnervation. The arm sling was weaned at 6 weeks, and gradual passive range of motion exercises were initiated. By 3 months postsurgery, the patient demonstrated excellent recovery. Neuropathic pain had resolved, and active range of motion improved to 150° forward flexion, 140° abduction, and 70° external rotation (Fig. 7). Strength in the deltoid and supraspinatus was restored, and she denied any residual pain or numbness. The final follow-up radiographs demonstrated a congruent glenohumeral joint with no evidence of subluxation (Fig. 8).

At 6-month follow-up, the American Shoulder and Elbow Surgeons (ASES) score improved to 76.7/100 (pain: 40/50, function: 36.7/50). Sub-score analysis revealed full recovery (3/3) in basic activities of daily living (ADLs), with partial recovery (2/3) in grooming and overhand throwing. Residual weakness (1/3) was noted in overhead reaching, heavy lifting, and sports. These findings, alongside a stable rotator cuff repair, suggest that the current functional status reflects the expected timeline of neurological reinnervation following C5-C6 plexitis.

## Discussion

The development of neurological complications following ARCR is an infrequent yet significant occurrence, with overall reported rates typically remaining below 1-2%.^17^^,^^19^ While peripheral nerve injuries commonly involve the median (39%), ulnar (17%), and axillary (14%) nerves,^19^ the presentation of a delayed-onset brachial plexitis is exceedingly rare. Inferior subluxation of the shoulder, secondary to deltoid atony, is a hallmark of axillary nerve palsy—a complication frequently reported after shoulder stabilization procedures. However, this case is uniquely characterized by its 3-week delayed presentation and a precipitating mechanism involving acute neck stretching, which deviates from the typical immediate post-operative neurovascular deficits.

While intraoperative factors such as traction, patient positioning, and fluid extravasation are established causes of iatrogenic neuropathy,^13^ the incidence of direct structural nerve damage has significantly declined with modern arthroscopic techniques.^13^ Consequently, when neurological deficits manifest with a delayed onset—precluding immediate surgical trauma—clinicians must investigate extraoperative etiologies. In the absence of immediate post-operative symptoms, the differential diagnosis shifts toward external factors such as prolonged immobilization, sling-induced pressure, or postural compression,^9^ suggesting that nerve injury may arise from passive post-operative management rather than active surgical intervention.

The pivotal diagnostic differentiator in this case was the distribution of muscle involvement identified via EMG. While an isolated axillary nerve injury would manifest as deficits confined to the deltoid and teres minor, our patient's EMG demonstrated acute denervation involving the deltoid, biceps brachii, and infraspinatus. Given that the biceps brachii and infraspinatus are primarily C5-C6 (innervated via the musculocutaneous and suprascapular nerves, respectively), these findings localized the lesion proximal to the terminal branches. This confirmed a diagnosis of incomplete brachial plexitis involving the upper trunk or C5-C6 roots. Although PMS is not yet a standardized diagnostic modality, it serves as a valuable adjunct to EMG in localizing proximal brachial plexus or nerve root injuries. PMS offers a unique advantage in its ability to provide supramaximal stimulation to deep neural structures that are often inaccessible or painful to stimulate electrically. While comprehensive data on its absolute sensitivity and specificity are still being established in the literature, studies have shown that PMS is highly effective in detecting early axonal loss and conduction blocks in proximal segments. In our case, the correlation between PMS findings and needle EMG increased the diagnostic certainty of a C5-C6/upper trunk localization. This pattern of multinerve involvement is highly characteristic of Parsonage-Turner syndrome or postsurgical brachial plexitis, as described by Feinberg et al,^4^ where the pathology is localized to the upper trunk or proximal roots rather than a single terminal branch. Identifying this more proximal pathology was paramount, as it ruled out a simple peripheral nerve palsy and pointed toward an inflammatory or traction-related root-level insult.

Regarding the underlying cause, the patient had an asymptomatic C-spondylosis and chronic degenerative changes at C5 on PMS. We hypothesize that the acute inflammation and soft tissue changes from the recent ARCR, combined with the patient's pre-existing root vulnerability, created a susceptible environment. Arthroscopy may precipitate symptoms by decompensating pre-existing, subclinical anatomical vulnerabilities, effectively unmasking previously asymptomatic conditions.^5^ Additionally, the forceful neck stretch may have acutely tethered or stretched the compromised C5-C6 roots, precipitating the symptomatic brachial plexitis and subsequent muscle weakness, leading to recurrent inferior subluxation. The potential for a “double-crush syndrome” in this patient cannot be overlooked. The relationship between cervical radiculopathy and brachial plexopathy is significant in this context, as pre-existing cervical nerve root irritation may lower the threshold for secondary injury to the brachial plexus.^23^ This theory suggests that a proximal neural compression—in this case, the mild C-spondylosis seen on PMS—renders the distal portion of the nerve more susceptible to injury from a secondary insult, such as post-operative inflammatory edema or mechanical stretching. Surgeons should be cognizant that patients with even subclinical cervical pathology may harbor a lower threshold for neurapraxia following shoulder procedures, necessitating careful intraoperative positioning and post-operative monitoring.

Beyond surgical positioning and mechanical strain, the interscalene block must be considered a potential causative or contributing factor in the development of this neurologic complication. Although interscalene block is a cornerstone of anesthesia in shoulder surgery, recent systematic reviews indicate that it carries a risk of up to 13% for prolonged post-operative neurologic symptoms.^1^ A critical aspect of interscalene block-related nerve injury is its delayed presentation or diagnosis, which is frequently reported^1^^,^^21^^,^^22^ and aligns with the 3-week symptomatic onset observed in our patient. These neurologic complications may arise from direct mechanical trauma, localized neurotoxicity, or post-block inflammatory changes. Furthermore, cases of severe and persistent nerve palsy following ultrasound-guided interscalene block have been documented in shoulder surgery, highlighting the vulnerability of the brachial plexus even when standard techniques are employed.^18^ In our case, it is plausible that the interscalene block induced a subclinical inflammatory state or “first hit” to the superior trunk, which was subsequently exacerbated and clinically unmasked by the mechanical neck stretch at three weeks.

The diagnostic algorithm for suspected nerve injury necessitates a rigorous clinical assessment, ideally supplemented by EMG within the first three weeks and cervical spine MRI when indicated.^3^ Pope et al emphasize that electrodiagnostic testing provides critical data beyond clinical examination, specifically by distinguishing inflammatory neuritis from compressive neuropathies. These modalities allow for precise localization of the lesion, characterization of axonal versus demyelinating pathology, and staging of denervation. Such objective findings are essential for guiding surgical decision-making and ruling out confounders like cervical radiculopathy.^14^

The majority of peripheral neuropathies following shoulder surgery resolve spontaneously with conservative management.^2^^,^^19^ Surgical intervention is typically reserved for cases of persistent, complete axillary nerve palsy that fail to respond to conservative management over a three-to-six-month period. As spontaneous resolution is observed in only 20% of such high-grade injuries, the vast majority of patients ultimately require operative reconstruction, most commonly via nerve transposition.^8^ Our patient, diagnosed with brachial plexitis, achieved full functional recovery by 3 months post-injury using a Bobath sling for immobilization and a conservative rehabilitation protocol. Although surgery remains an option for persistent neurological deficits, this case provides a strong example of successful non-operative management even for proximal plexus injuries. This underscores the importance of a thorough neurological workup to avoid unnecessary surgical intervention for what may be a transient plexopathy.

There are certain limitations to this report. As a single case study, the findings cannot be generalized to all patients undergoing ARCR. Furthermore, while the temporal relationship between the neck stretch and the onset of symptoms is compelling, other perioperative factors such as the duration of arm traction might have contributed to the neural compromise. Future prospective studies are required to further elucidate the relationship between pre-existing cervical spondylosis and the risk of delayed brachial plexitis.

## Conclusion

This case highlights a rare but significant occurrence of delayed-onset incomplete brachial plexitis (C5-C6) following ARCR. While post-operative neurological deficits are often attributed to peripheral nerve injury, clinicians must remain vigilant for proximal plexopathies that may present weeks after the initial procedure. Furthermore, this case suggests that pre-existing cervical spondylosis may act as a predisposing factor for neural compromise when combined with post-operative inflammatory changes and mechanical triggers. Surgeons should consider this potential risk when managing patients with known cervical pathology undergoing complex shoulder arthroscopy.

## Declaration of generative AI and AI-assisted technologies in the writing process

Google Gemini was used for language and structural refinement. All authors reviewed/approved the final text and take full responsibility for its accuracy.

## Disclaimers:

Funding: No funding was disclosed by the authors.

Conflicts of interest: The authors, their immediate families, and any research foundations with which they are affiliated have not received any financial payments or other benefits from any commercial entity related to the subject of this article.

Patient consent: Obtained.
